# Upregulation of Synuclein-*γ* and Snai1 Contributes to Poor Clinical Prognosis in Oral Squamous Cell Carcinoma Patients

**DOI:** 10.1155/2022/6534626

**Published:** 2022-04-07

**Authors:** Jie Yang, Yangyang Pan, Lu Peng, Licui Zhang, Juan Zhao, Zhihong Zheng, Jun Zheng, Xiaoli Xu, Yan Zeng

**Affiliations:** ^1^Key Laboratory of Xinjiang Endemic and Ethnic Diseases, School of Medicine, Shihezi University, Shihezi, Xinjiang, China; ^2^Department of Laboratory, The First Affiliated Hospital, School of Medicine, Shihezi University, Shihezi, Xinjiang, China; ^3^Department of Stomatology, The First Affiliated Hospital, School of Medicine, Shihezi University, Shihezi, Xinjiang, China; ^4^Department of Hematology, The First People's Hospital of Foshan, Foshan, Guangdong, China

## Abstract

Synuclein-*γ* (SNCG) and Snai1 play an important role in the occurrence and development of different types of malignant tumors. However, the association between SNCG and Snai1 and the effect of their combination on oral squamous cell carcinoma (OSCC) are unknown. The purpose of this study was to assess the expression of SNCG and Snai1 in OSCC tissues and their role in the genesis, development, diagnosis, and prognosis of OSCC. In this study, we first analyzed the Gene Expression Omnibus (GEO) database to determine the expression of SNCG and Snai1 in OSCC. And we also evaluated the correlation between the expression of SNCG and Snai1 and clinical pathological parameters in OSCC from The Cancer Genome Atlas (TCGA) database. Then, the expression of SNCG and Snai1 in OSCC and its adjacent tissues in our experimental cohort were detected by qRT-PCR, Western blot, and immunohistochemistry, and the relationship between their expression and clinical pathological parameters were analyzed. At the same time, the correlation between the expression of SNCG and Snai1 was analyzed from the TCGA, GEO database, and our experimental cohort. Next, the ROC curves were constructed to explore the diagnostic value of SNCG and Snai1 in OSCC. Finally, the survival curves were drawn, and the univariate and multivariate Cox regression analyses were performed to determine the prognostic value of SNCG and Snai1 in OSCC. The study found that SNCG and Snai1 were highly expressed in OSCC tissues. The expression of SNCG was related to the differentiation of OSCC, while that of Snai1 was related to the T stage, lymph node metastasis, clinical stage, and differentiation. Besides, the expression of SNCG in OSCC was positively correlated with that of Snai1. In addition, we also found that SNCG and Snai1 could well distinguish OSCC patients from normal people; especially, the combined diagnosis of SNCG and Snai1 had a better effect, with a specificity up to 96.67%. Moreover, SNCG-negative/Snai1-negative OSCC patients had the best prognosis. Multivariate analysis displayed that SNCG-positive expression was an independent risk factor for prognosis in OSCC patients. The results of this study strongly suggested that SNCG and Snai1 might have a cooperative effect in the occurrence and development of OSCC. They may become new markers for the diagnosis and prognosis of OSCC.

## 1. Introduction

Oral cavity cancer is one of the most common malignant tumors in the head and neck, with 170,000 deaths and over 350,000 new cases in 2018 alone worldwide [1, 2]. Oral squamous cell carcinoma (OSCC) always happens in the gingiva, hard palate, tongue, buccal mucosa, and lip, accounting for 95% of all oral cavity cancers [3]. At present, it is found that the peak incidence of OSCC is between 45 and 75 years old [4, 5]. In recent years, with the improvement of treatment methods, the quality of life of invalids with OSCC has been greatly improved. However, the five-year survival rate of the patients is not significantly improved and is still maintained at about 50-60%, but the five-year survival rate of early OSCC patients after treatment is as high as 85.4% [6]. Therefore, for OSCC, finding tumor markers for diagnosis and prognosis is of great significance for the treatment of tumors and also the development direction in the future.

Synuclein-*γ* (SNCG) is a member of the small protein synuclein family, which is abnormally expressed in many tumor tissues, but almost no expression in matched noncancerous tissues [7–13]. In recent years, researchers have found that SNCG is associated with the occurrence and progression of gallbladder cancer and colorectal cancer, and SNCG is highly expressed in liver metastatic cells of colon cancer [14–16]. In addition, when SNCG is overexpressed, it promotes the expression of MMP-2 and MMP-9, which further promotes cell metastasis [17]. The above studies have shown that SNCG is closely related to the occurrence and development of tumors, and it deserves to be further studied.

Snai1 is a zinc finger transcription factor that accelerates cell survival and metastasis by inducing EMT [18]. Snai1 inhibits E-cadherin and other genes that maintain epithelial phenotype and increases vimentin and N-cadherin that maintain interstitial phenotype [19]. Previous studies have pointed out that the high expression of Snai1 has a significant correlation with the metastasis, recurrence, and poor prognosis of a variety of epithelial-derived malignant tumors, including colorectal cancer, lung cancer, and breast cancer [20–23]. The above reports suggest that Snai1 plays an important role in tumor occurrence and development, but the clinical application of Snai1 in OSCC is still limited, which deserves further research and attention.

Our previous study has found that the levels of SNCG in saliva and serum of OSCC patients are higher than those of patients with oral potential malignant diseases and healthy controls and are related to the differentiation of OSCC [24, 25]. On the basis of previous studies, we intended to further explore the expression of SNCG in OSCC tissues and its value in the diagnosis and prognosis of OSCC. Moreover, SNCG promotes the migration, invasion, and metastasis of tumor cells. SNCG downregulates the expression levels of epithelial markers E-cadherin and ZO-1, upregulates the expression of mesenchymal marker vimentin, and regulates the progress of EMT. However, the relationship between SNCG and EMT marker Snai1 has not been reported yet. Notably, Snai1, as one of the molecules of EMT, can drive EMT by itself and plays an indispensable role in regulating other core transcription factors of EMT. Both SNCG and Snai1 can regulate EMT-related molecules, and Snai1 is also an EMT molecule. Therefore, we wonder if there is a correlation between SNCG and Snai1.Therefore, the purpose of this study is to investigate the expression and correlation of SNCG and Snai1 in OSCC tissues and the value of combined detection of SNCG and Snai1 in diagnosis and prognosis of OSCC.

## 2. Material and Methods

### 2.1. Bioinformatics Analysis

The mRNA expression of SNCG and Snai1 in HNSCC and corresponding adjacent tissues and the clinicopathological parameters of HNSCC patients were downloaded from TCGA (http://ualcan.path.uab.edu/analysis.html) database. And the mRNA expression of SNCG and Snai1 in OSCC and normal tissues was downloaded from GEO (http://www.ncbi. http://nlm.nih.gov/geo) database. A total of 546 cases were obtained from the TCGA database, including 44 cases of adjacent tissues and 502 cases of cancer tissues (one of the cancer tissue samples was the same patient's repeated sampling). Four types of samples were excluded, including throat, tonsil, hypopharynx, and oropharynx. The remaining 331 OSCC samples and 32 adjacent samples include 126 cases of oral tongue samples, 72 cases of oral cavity samples, 60 cases of the floor of mouth samples, 23 cases of the base of tongue samples, 22 cases of buccal mucosa samples, 18 cases of alveolar ridge samples, 7 cases of hard palate samples, and 3 cases of lip samples. In the meantime, 45 cases of normal tissues and 167 cases of OSCC tissues from the data set GSE30784 of GEO database were recruited in this study, which included two ID of 208584 and 209877 to describe SNCG and Snai1 mRNA expression.

### 2.2. Patients and Samples

This study was approved by the ethics committee of the First Affiliated Hospital of Shihezi University School of Medicine. All patients who provided tissue samples provided informed consent for their data used in this study. All OSCC tissues and adjacent normal tissues were from OSCC patients with complete clinical data and pathological diagnosis in the First Affiliated Hospital of Shihezi University School of Medicine from 2012 to 2020, including 94 OSCC tissue samples and 30 adjacent normal tissue samples. All tumors were classified according to the eighth edition of AJCC Cancer Staging Manual, 70 cases were stage I+II, and 24 cases were stage III+IV. The presence of lymph node metastasis was identified by histological examination. The overall survival (OS) was determined by the time interval between the treatment of the disease and death from any cause. The disease-free survival (DFS) was measured by the time interval between treatment of the disease and tumor recurrence or death caused by the tumor. All patients underwent radical surgery, and no one received chemotherapy, radiotherapy, or biotherapy before surgery. The follow-up data for all patients were obtained by interview or telephone for 8 years (medium: 63 months and 14–94 months).

### 2.3. qRT-PCR

Total RNA was extracted from clinical specimens (including 14 pairs of OSCC and paired adjacent normal tissues) using TRIzol reagent (Invitrogen, Carlsbad). According to the manufacturer's instructions, PrimeScript™ RT kit and cDNA Synthesis kit (Takara, Glen Burnie) were used to reverse transcribe RNA (500 ng). SYBR Green qPCR Master Mix (TOYOBO) was used to quantify the expression of SNCG and Snai1. The 2-*Δ*Ct method was used to calculate the relative amount of SNCG and Snai1. The primers used were as follows: SNCG, forward: 5′-CAAGAAGGGCTTCTCCATCG CCAAGG-3′, reverse: 5′-CCTCTTTCTCTTTGGATGCCACACCC-3′, and Snai1, forward: 5′-C CACACTGGCGAGAAG-3′, reverse: 5′-AGAAGGTCCGAGCACA C-3′.

### 2.4. Western Blot

The frozen tissues (including 6 pairs of OSCC and paired adjacent normal tissues) were dissolved in RIPA buffer containing 1% PMSF (Sigma, St. Louis). 15% SDS-PAGE gels were used for electrophoresis of lysates, and lysates were transferred onto PVDF membranes (Merck Micropore). After blocking with 5% nonfat milk, the membranes were incubated with anti-SNCG antibody (1 : 250; Peking University Cancer Hospital and Institute, China), anti-Snai1 antibody (1 : 500; Cell Signaling Technology, USA), and anti-*β*-actin antibody (1 : 5000; ZSGB-BIO, China) at 4°C overnight. The membranes were treated with peroxidase-conjugated IgG antibody for 2 hours at room temperature. The protein bands were revealed by detection of enhanced chemiluminescence reagent (Pierce).

### 2.5. Immunohistochemistry Staining

The expression of SNCG and Snai1 was detected by immunohistochemical envision method. After baking, the paraffin sections were dewaxed and rehydrated by xylene and a series of alcohol. The peroxidase was blocked by incubation with 3% hydrogen peroxide. Then, thermal repair of the antigen was performed in EDTA. After blocking with 10% goat serum, anti SNCG McAb No. 1 (Peking University Cancer Hospital and Institute) and anti-Snai1 antibody (Abcam, USA) were dropped at 4°C for overnight incubation, and the secondary antibody (Dako Cytomation, Cambridge, UK) was added after washing with PBS. Then DAB staining, hematoxylin contrast staining and neutral glue patch were performed.

The immunohistochemical results were randomly double-blind read and scored by two experienced pathologists. Five regions were selected for each sample to evaluate the results. According to staining intensity and percentage of positive cells, the criteria are as follows: The staining intensity includes absent (0), weak (1), moderate (2), and strong (3). The percentage of stained cells to evaluate the staining range includes 0%-5% (0), 6%-25% (1), 26%-50% (2), 51%-75% (3), and 76%-100% (4). The final score was based on the product of the staining intensity and range scores: 0–1 was negative (−), 2–3 was weak positive (1+), 4–6 was moderate positive (2+), and 8–12 was strong positive (3+). Cases were divided into two categories according to SNCG and Snai1 expression levels: negative expression (−/1+) and positive expression (2+/3+).

### 2.6. Statistical Analysis

All data were analyzed by SPSS 23.0 statistical software. The Spearman rank correlation analysis was used to analyze the correlation between SNCG and Snai1 expression in TCGA, GEO database, and our experimental cohort. The chi-square test was used to compare the count data between groups. *T* test was used to analyze the mRNA expression of SNCG and Snai1 in OSCC and normal tissues in GEO database and also used to analyze the relationship between the mRNA expression of SNCG and Snai1 and clinicopathological parameters of OSCC in TCGA database. Paired *T* test was used to analyze the mRNA expression of SNCG and Snai1 in OSCC tissues and their paired adjacent normal tissues. Receiver operating characteristic (ROC) curves were created by SPSS software to evaluate the diagnostic values of SNCG and Snai1, and the cut-off points of SNCG and Snai1 were determined by the Youden index. The survival curves were constructed by the Kaplan-Meier method, and the significance of difference was examined by the log-rank test. The Cox proportional hazard regression model was used for univariate and multivariate analyses. All data were calculated with 95% confidence intervals (95% CI). All data were statistically significant when *P* < 0.05.

## 3. Results

### 3.1. The Expression of SNCG/Snai1 in OSCC and Their Relationship with Clinicopathological Features in Database

**Figure 1:**
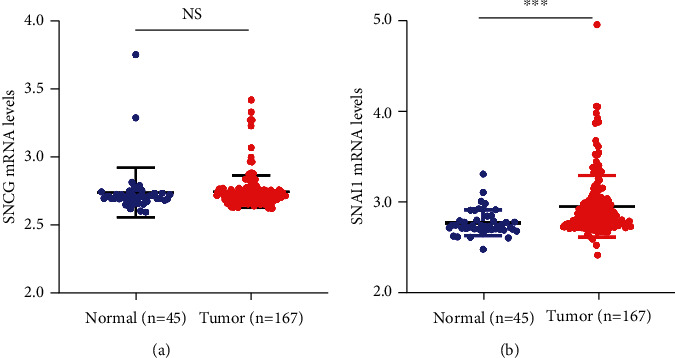
The expression levels of SNCG and Snai1 in OSCC and normal tissues in GEO database. (a) There was no significant difference in the mRNA expression of SNCG between 167 OSCC tissues (2.742 ± 0.009) and 45 normal tissues (2.736 ± 0.028) (*P* > 0.05). (b) Snai1 was dramatically upregulated in 167 OSCC tissues (2.941 ± 0.027) compared with 45 normal tissues (2.758 ± 0.022) in the GEO database (*P* < 0.05). *P* < 0.05 is considered statistically significant. NS: not statistically different. ^∗∗∗^*P* < 0.001.

Based on the GEO database, we analyzed the mRNA expression of SNCG and Snai1 in OSCC and normal tissues. As shown in Figure 1, there was no significant difference in the mRNA expression of SNCG between OSCC and normal tissues (Figure 1(a)), but the mRNA expression of Snai1 in OSCC tissues was higher than that in normal tissues (Figure 1(b)).

**Figure 2:**
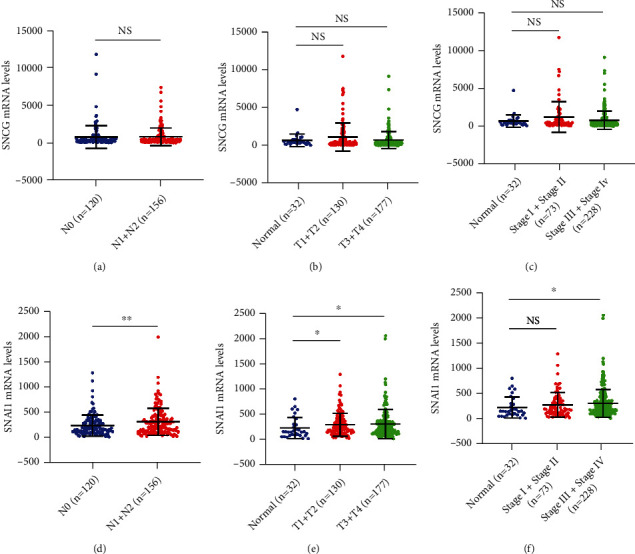
The relationship between SNCG and Snai1 expression and clinical features in OSCC patients from TCGA database. (a–c) Comparison of expression of SNCG under different clinicopathological parameters in TCGA database: (a) lymph node metastasis, (b) T stage, and (c) clinical stage. (d–f) Comparison of expression of Snai1 under different clinicopathological parameters in TCGA database: (d) lymph node metastasis, (e) T stage, and (f) clinical stage. NS: not statistically different. ^∗^*P* < 0.05 and ^∗∗^*P* < 0.01.

Furthermore, we explored the role of SNCG and Snai1 in the development of OSCC. We analyzed the correlation between the expression of SNCG and Snai1 and clinical parameters of OSCC in the TCGA database and focused on the correlation between the expression of SNCG and Snai1 and lymph node metastasis, T stage, and clinical stage. As shown in Figure 2, the mRNA expression of SNCG in OSCC was not related to lymph node metastasis, T stage, and clinical stage (Figures 2(a)–2(c)), while the mRNA expression of Snai1 in OSCC was related to lymph node metastasis, T stage, and clinical stage (Figures 2(d)–2(f)).

### 3.2. The Expression of SNCG/Snai1 in OSCC and Their Relationship with Clinicopathological Features in our Experimental Cohort

**Figure 3:**
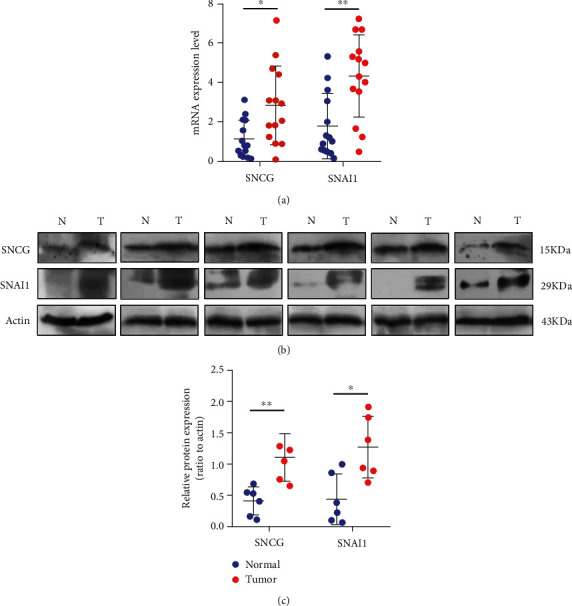
SNCG and Snai1 are upregulated in OSCC. (a) The mRNA expression levels of SNCG and Snai1 in OSCC tissues and their paired adjacent normal tissues by qRT-PCR. (b) The protein expression levels of SNCG and Snai1 in OSCC tissues and their paired adjacent normal tissues by Western blot. N: normal; T: tumor. Three independent experiments were conducted for each assay. (c) Quantification of SNCG and Snai1 levels in Western blot. ^∗^*P* < 0.05 and ^∗∗^*P* < 0.01.

Based on the analysis results in the database, the mRNA expression of SNCG and Snai1 in 14 pairs of OSCC tissues and their paired adjacent normal tissues was analyzed by qRT-PCR, as shown in Figure 3(a). In addition, the protein expression of SNCG and Snai1 in 6 pairs of OSCC tissues and their paired adjacent normal tissues was analyzed by Western blot, as shown in Figure 3(b).  Figure 3(c) shows the quantification of the Western blot bands. The above results showed that both mRNA and protein expression levels of SNCG and Snai1 in OSCC tissues were higher than those in their paired adjacent normal tissues.

**Figure 4:**
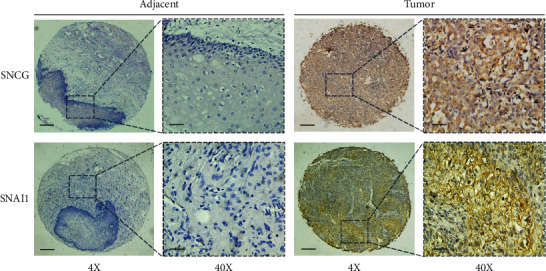
The immunohistochemistry (IHC) staining for SNCG and Snai1 in OSCC tissues and adjacent normal tissues (scale bar, 50 *μ*m).

To confirm the above results, we made the immunohistochemical staining on 94 cases of OSCC tissues and 30 cases of adjacent normal tissues and detected the SNCG and Snai1 expression. We divided all samples into two subgroups according to SNCG and Snai1 protein expression levels (0, 1+, 2+, and 3+): negative expression (0/1+) and positive expression (2+/3+). As shown in Figure 4, both SNCG and Snai1 were expressed in the cytoplasm and nucleus, while SNCG was mainly located in the cytoplasm. In 94 cases of OSCC, the positive rates of SNCG and Snai1 were 61/94 and 59/94, respectively. In 30 cases of adjacent normal tissues, the positive rates of SNCG and Snai1 were 7/30 and 5/30, respectively (Table 1). Immunohistochemical results showed that both SNCG and Snai1 expression in OSCC tissues were higher than that in adjacent normal tissues (*P* < 0.05). These results strongly indicated that SNCG and Snai1 were upregulated in human OSCC tissues.

To study the role of SNCG and Snai1 in OSCC patients, the association between SNCG and Snai1 expression and patients' clinicopathological parameters was studied.  Table 2 shows that SNCG expression was related to the differentiation of OSCC (*P* < 0.05). The expression of Snai1 was related to the T stage, clinical stage, lymph node metastasis, and tumor differentiation (*P* < 0.05). These results were consistent with the TCGA database.

### 3.3. The Correlation between SNCG and Snai1 Expression in OSCC

**Figure 5:**
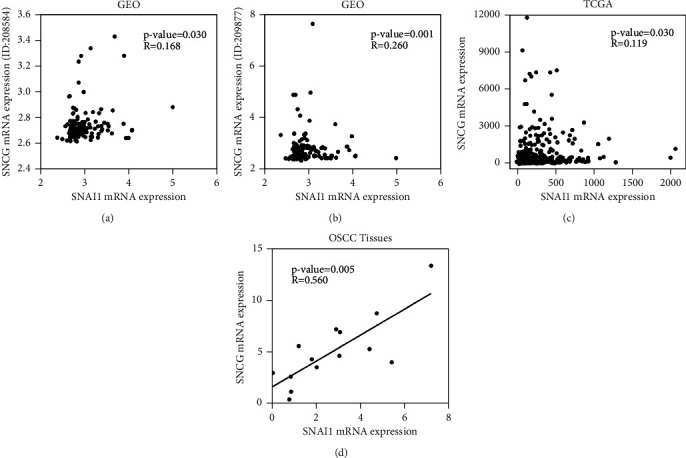
Association with SNCG and Snai1 in patients with OSCC. (a–c) Correlation analysis of SNCG and Snai1 mRNA expression in OSCC based on the ID of 208584 (a) and 209877 (b) from the data set GSE30784 of GEO database and TCGA database (c). (d) Bivariate correlation analyses showing a positive correlation between SNCG and Snai1 expression in patients with OSCC, *P* < 0.05, Spearman's test.

The above results indicated that both SNCG and Snai1 were highly expressed in OSCC. We speculated that there might be a correlation between SNCG and Snai1 in OSCC. Therefore, we went on to analyze the correlation expression of SNCG and Snai1 in OSCC through GEO and TCGA databases (Figures 5(a)–5(c)). It was found that SNCG and Snai1 were positively and linearly correlated in patients with OSCC. Consistently, qRT-PCR analysis showed that SNCG mRNA levels were positively correlated with Snai1 mRNA levels in OSCC patients from our experimental cohort (Figure 5(d)). In addition, the Spearman rank correlation analysis was performed on the immunohistochemical results, which was consistent with the results of mRNA analysis. The protein expressions of SNCG and Snai1 were also positively correlated (*r* = 0.586, *P* < 0.001) (Table 3).

### 3.4. The Diagnostic Value of SNCG and Snai1 in OSCC

**Figure 6:**
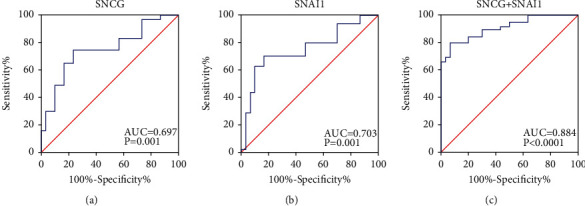
The diagnostic value of SNCG and Snai1 in OSCC. The ROC curves of SNCG (a), Snai1 (b), and the combination of the two (c) in distinguishing OSCC patients from normal subjects. The area under the ROC curves (AUCs) was computed to compare the capacity of SNCG and Snai1 to distinguish OSCC from normal subjects.

The ROC curves were constructed to explore the diagnostic value of SNCG and Snai1 in OSCC. As shown in Figure 6, the AUC value of SNCG for diagnosing OSCC was 0.697, the cut-off value was 3.5, sensitivity and specificity were 64.89% and 76.67%, respectively, and 95% CI was 0.595 to 0.799 (Figure 6(a)) (Table 4). The AUC value of Snai1 for diagnosing OSCC was 0.703, the cut-off value was 3.5, sensitivity and specificity were 62.77% and 83.33%, respectively, and 95% CI was 0.603 to 0.804 (Figure 6(b)) (Table 4). Data of the combined SNCG and Snai1 were established by a logistic regression model [Logit (*P*) = 0.214 − 0.166 × SNCG + 0.404 × Snai1]. It should be noted that, when SNCG and Snai1 were combined to diagnose OSCC, the AUC was 0.884, the sensitivity and specificity were 65.96% and 96.67%, respectively, and the 95% CI was 0.826 to 0.942, which was superior to the detection of single index significantly (Figure 6(c)) (Table 4).

### 3.5. The Positive Expression of SNCG and Snai1 in OSCC Patients Has a Poor Prognosis

**Figure 7:**
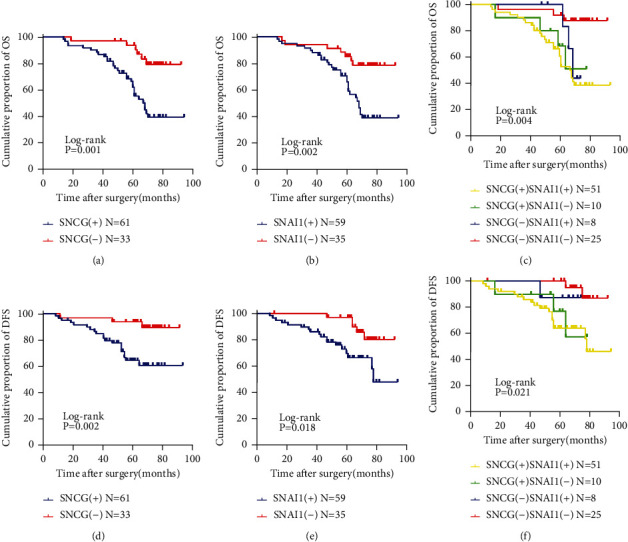
Survival analysis of patients with OSCC with positive or negative SNCG and Snai1 expression. (a) OSCC patients with positive expression of SNCG had shorter OS rate (log-rank test *X*^2^ = 12.103, *P* = 0.001). (b) OSCC patients with positive expression of Snai1 had shorter OS rate (log-rank test *X*^2^ = 9.375, *P* = 0.002). (c) OSCC patients with positive expression of both SNCG and Snai1 had the shortest OS (log-rank test *X*^2^ = 13.570, *P* = 0.004). (d) OSCC patients with positive expression of SNCG had shorter DFS rate (log-rank test *X*^2^ = 9.430, *P* = 0.002). (e) OSCC patients with positive expression of Snai1 had shorter DFS rate (log-rank test *X*^2^ = 5.640, *P* = 0.018). (f) OSCC patients with positive expression of both SNCG and Snai1 had the shortest OS (log-rank test *X*^2^ = 9.788, *P* = 0.021).

To explore the relationship between the expression of SNCG and Snai1 and the prognosis of OSCC patients, the Kaplan-Meier survival curves were applied. Survival results showed that the OS of patients with positive SNCG/Snai1 expression was worse than those with negative SNCG/Snai1 expression (Figures 7(a) and 7(b)). Similarly, the DFS of patients with positive SNCG/Snai1 expression was worse than those with negative SNCG/Snai1 expression (Figures 7(d) and 7(e)). In addition, it was found that OSCC patients had the worst OS and DFS when the expressions of SNCG and Snai1 were positive at the same time; on the contrary, SNCG and Snai1 negative patients had the best OS and DFS (Figures 7(c) and 7(f)).

**Figure 8:**
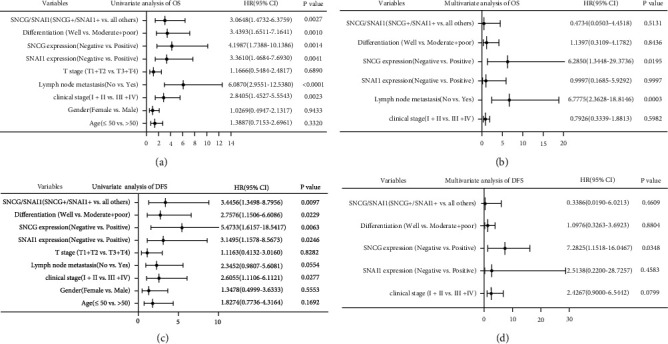
The relationship between SNCG and Snai1 expression and prognosis of OSCC. (a) Univariate and (b) multivariate Cox proportional hazard analysis for OS of OSCC. (c) Univariate and (d) multivariate Cox proportional hazard analysis for DFS of OSCC.

To further explore the risk factors of OS and DFS in patients with OSCC, we made univariate and multivariate analyses using the Cox proportional hazard regression model. Univariate analysis showed that clinical stage (HR = 2.8405), lymph node metastasis (HR = 6.0870), differentiation (HR = 3.4393), SNCG expression (HR = 4.1987), Snai1 expression (HR = 3.3610), and SNCG/Snai1 (HR = 3.0648) were risk factors of OS (Figure 8(a)). Multivariate analysis indicated that lymph node metastasis (HR = 6.7775) and SNCG expression (HR = 6.2850) were independent prognostic factors of OS in OSCC patients (Figure 8(b)). In addition, univariate analysis showed that clinical stage (HR = 2.6055), differentiation (HR = 2.7576), SNCG expression (HR = 5.4733), Snai1 expression (HR = 3.1495), and SNCG/Snai1 (HR = 3.4456) were risk factors of DFS (Figure 8(c)). Multivariate analysis indicated that SNCG expression (HR = 7.2825) was an independent prognostic factor of DFS in OSCC patients (Figure 8(d)).

## 4. Discussion

OSCC, with the highest incidence of oral cancer, has become one of the leading causes of death in China due to its early and extensive metastasis. At present, its pathogenesis is still unclear. Smoking, alcohol consumption, human papillomavirus (HPV) infection, betel nut chewing, and immunodeficiency are all risk factors for this disease [26, 27]. At present, the main treatment methods of OSCC are surgical resection, chemotherapy and radiotherapy, or the combination of these three methods [28]. Although the treatment methods have been improved continuously, the five-year survival rate has not increased significantly in recent years due to the abundant blood vessels and nerves in oral and maxillofacial tissues, high incidence of cervical lymph node metastasis and invasion, and poor prognosis. The 5-year survival rate of patients with advanced tumor or tumor recurrence is lower [29, 30]. Therefore, it is of great significance for patients with OSCC to find biomarkers for the diagnosis and prognosis of OSCC. At present, a large number of scholars have committed to the study of OSCC biomarkers. Zhong et al. [31] found that Cyfra 21-1 could be used as a diagnostic marker of OSCC. Feng et al. [32] also found that SCCAg could be used as a diagnostic marker of OSCC. Another study found that PMS2 was a potential prognostic marker of OSCC [33]. Although researchers have found that many genes play key roles in the formation of OSCC. But so far, no reliable biomarker has been found to use as an indicator for the early diagnosis and prognosis evaluation of OSCC.

SNCG was first found in human breast cancer cDNA library, and it has been established that it could promote the metastasis of breast cancer [34]. Recently, abnormal high expression of SNCG has been found in ovarian cancer, colorectal cancer, and other malignant tumors [35, 36], especially high expression in advanced tumors, suggesting that SNCG has lost its original tissue specificity in the process of tumor development and is expected to be an effective tumor marker. In this study, we explored the expression and role of SNCG in OSCC. First, we detected the mRNA and protein expression of SNCG in OSCC and found that SNCG was highly expressed in OSCC tissues, which was consistent with our previous study [24, 25]. And our results were consistent with those reported in multiple articles, which indicate that SNCG was highly expressed in a variety of tumors [9, 37, 38]. However, our experimental results were inconsistent with those in the GEO database, and the results from the GEO database indicated that there was no difference in the SNCG expression between OSCC and normal tissues. Our previous study found that the expression of SNCG in OSCC was related to ethnic differences [25]. Therefore, the difference between our experimental results and database results may be due to different sample sources and ethnic differences, which may require more samples to test. At the same time, we analyzed the correlation between the expression of SNCG in OSCC and clinicopathological features and found that the expression of SNCG was related to differentiation, which was consistent with our previous study [24, 25]. Next, we explored the diagnostic value of SNCG in OSCC tissues. The results showed that SNCG could distinguish OSCC patients from normal people, which was consistent with the report by Liu et al. [39], who found that urine SNCG could distinguish bladder cancer from urinary system diseases and could be used as a marker for the diagnosis of bladder cancer. In addition, our previous study has shown that serum SNCG and SCCAg have good diagnostic value for OSCC, and the combination of these two factors has a higher diagnostic efficiency in distinguishing OSCC from normal people, with an AUC of 0.998, a sensitivity of 97.7%, and a specificity of 98.85% [25]. In the present study, when SNCG and Snai1 were combined to diagnose OSCC, the AUC was 0.884, and the sensitivity and specificity were 65.96% and 96.67%, respectively. The combined diagnosis of SNCG and SCCAg was superior to that of SNCG and Snai1. The possible reason is that the diagnostic level of serum is better than that of tissue. We can study the diagnostic capability of SNCG and Snai1 in the level of serum in future studies, and we can also detect the diagnostic value of the combination of these three factors in OSCC at the serum level. It is well known that differentiation is closely related to prognosis, based on our result of the correlation between SNCG expression and differentiation. Therefore, we evaluated the role of SNCG in the prognosis of OSCC. Survival analysis showed that OSCC patients with high SNCG expression had a poor prognosis. The univariate Cox regression analyses indicated that SNCG expression was a risk factor for OS and DFS in OSCC patients, and the multivariate Cox regression analyses also indicated that SNCG was an independent prognostic factor for OS and DFS in OSCC patients. These were consistent with the report by Zhang et al. [40] in 2020; the study reported that SNCG was an independent prognostic factor for ovarian cancer. These were also consistent with the report by Paulette et al. [36], who demonstrated that the overexpression of SNCG appeared to be a prognostic biomarker for patients with endometrial cancer.

As a transcription factor, Snai1 can bind to the promoter sequence of downstream target genes and play a role in transcriptional regulation. Snai1 is overexpressed in a variety of tumors, and its overexpression induces EMT of tumor cells and promotes metastasis and invasion [41]. This study found that the expression of Snai1 in OSCC tissues was upregulated compared with that in adjacent tissues, which was consistent with the report by Peng et al. [42], who found that the expression of Snai1 was upregulated in oral submucosal fibrosis (OSF) samples. At the same time, our study showed that the expression of Snai1 in the OSCC tissue was related to T stage, clinical stage, lymph node metastasis and differentiation. The study of Song et al. [43] found that the positive expression of Snai1 protein in esophageal squamous cell carcinoma (ESCC) was related to T stage, lymph node metastasis, and TNM stage, which was consistent with our results. Wang et al. [44] also reported that the increased Snai1 expression was significantly associated with lymph node metastasis, clinical stage, and poor prognosis in colorectal cancer (CRC) patients. Next, we analyzed the diagnostic value of Snai1 in OSCC and found that Snai1 could distinguish patients with OSCC from normal people. This study also found that there was a positive correlation between SNCG and Snai1 expression, suggesting that SNCG and Snai1 may therefore have a cooperative effect in the occurrence and progression of OSCC. Moreover, more and more reports indicated that the combined diagnosis was more effective than the single diagnosis. Therefore, we analyzed the ability of SNCG in combination with Snai1 to diagnose OSCC. The results showed that the diagnostic efficiency of combined diagnosis was higher than those of the two single diagnosis on OSCC. In addition, we found that OSCC patients with high expression of Snai1 had poor prognosis; this was consistent with the report by Fang and Ding [45], who demonstrated that Snai1 could be used as a biomarker for the prognosis of gastrointestinal cancer. More interestingly, the prognosis of patients was the worst when both SNCG and Snai1 were positive and the best when both were negative. This was consistent with the report by Tian et al. [46], in which the combined application of Snai1 and E-cadherin outperformed the individual indicators in predicting OS in patients with cervical cancer.

In conclusion, our study has demonstrated that both SNCG and Snai1 are highly expressed in OSCC tissues and participate in the occurrence and development of OSCC. This study is the first to report the correlation between SNCG and Snai1 and also the first to report the value of the combination of SNCG and Snai1 in the diagnosis and prognosis of OSCC. It has important theoretical reference value for the clinical diagnosis, prognosis prediction, and targeted therapy of OSCC. However, we are unable to provide more research results to prove the cellular biological functions and related mechanisms of SNCG and Snai1 in OSCC. Therefore, more studies are needed to support our results and assumptions.

## 5. Conclusions

To sum up, the data obtained by mRNA and protein detection methods have confirmed that SNCG and Snai1 are overexpressed in OSCC. The combination of SNCG and Snai1 can be a good marker for the early diagnosis and prognosis prediction of OSCC. More experiments are ongoing to answer remaining questions about its cellular functions and molecular mechanisms.

## Figures and Tables

**Table 1:** The expression of SNCG and Snai1 in OSCC and adjacent tissues.

Tissues	*n*	SNCG		*χ* ^2^	*P*	Snai1		*χ* ^2^	*P*
		Positive	Negative			Positive	Negative		
Adjacent	30	7	23	15.86	*P* ≤ 0.001	5	25	17.55	*P* ≤ 0.001
OSCC	94	61	33			59	35		

**Table 2:** The relationship between the expression of SNCG and Snai1 and the clinicopathological features of OSCC patients.

Characteristics	Cases (*n*)	SNCG		Snai1			
		Negative (%)	Positive (%)	*P*	Negative (%)	Positive (%)	*P*
*Age*							
≤50	44	18 (40.9)	26 (59.1)	0.269	19 (43.2)	25 (56.8)	0.263
>50	50	15 (30.0)	35 (70.0)		16 (32.0)	34 (68.0)	
*Gender*						
Male	71	23 (32.4)	48 (67.6)	0.333	26 (36.6)	45 (63.4)	0.829
Female	23	10 (43.5)	13 (56.5)		9 (39.1)	14 (60.9)	
*T stage*						
T1+T2	74	23 (31.1)	51 (68.9)	0.116	32 (43.2)	42 (56.8)	0.020
T3+T4	20	10 (50.0)	10 (50.0)		3 (15.0)	17 (85.0)	
*Clinical stage*						
I+II	70	28 (40.0)	42 (60.0)	0.090	32 (45.7)	38 (54.3)	0.004
III+IV	24	5 (20.8)	19 (79.2)		3 (12.5)	21 (87.5)	
*Lymph node metastasis*						
No	60	25 (41.7)	35 (58.3)	0.077	32 (53.3)	28 (46.7)	*P* ≤ 0.001
Yes	34	8 (23.5)	26 (76.5)		3 (8.8)	31 (91.2)	
*Differentiation*							
High	45	28 (62.2)	17 (37.8)	*P* ≤ 0.001	29 (64.4)	16 (35.6)	*P* ≤ 0.001
Middle and low	49	5 (10.2)	44 (89.8)		6 (12.2)	43 (87.8)	

**Table 3:** The relationship between SNCG and Snai1 expression in OSCC.

Snai1	SNCG		*r*	*P*
	Positive	Negative		
Positive	51	8	0.586	*P* ≤ 0.001
Negative	10	25		

**Table 4:** The diagnostic value of SNCG and Snai1 in OSCC.

Indicators	AUC	95% CI	SE	Sensitivity (%)	Specificity (%)	PPV (%)	NPV (%)	Youden index value	P value
SNCG	0.697	0.595-0.799	0.052	64.89	76.67	89.71	41.07	0.416	0.001
Snai1	0.703	0.603-0.804	0.051	62.77	83.33	92.19	41.67	0.461	0.001
SNCG+Snai1	0.884	0.826-0.942	0.030	65.96	96.67	98.41	47.54	0.626	<0.0001

AUC: area under the curve; 95% CI: 95% confidence interval; SE: standard error; PPV: positive predictive value; NPV: negative predictive value.

## Data Availability

The data used to support the findings of this study are included within the article.
